# Comparative phylogeography of the plateau zokor (*Eospalax baileyi*) and its host-associated flea (*Neopsylla paranoma*) in the Qinghai-Tibet Plateau

**DOI:** 10.1186/s12862-014-0180-5

**Published:** 2014-08-17

**Authors:** Gonghua Lin, Fang Zhao, Hongjian Chen, Xiaogong Deng, Jianping Su, Tongzuo Zhang

**Affiliations:** 1 Key Laboratory of Adaptation and Evolution of Plateau Biota, Northwest Institute of Plateau Biology, Chinese Academy of Sciences, Xining 810008, China; 2 University of Chinese Academy of Sciences, Beijing 100049, China; 3 Qinghai Institute for Endemic Disease Prevention and Control, Xining 811602, China

**Keywords:** Zokor, Flea, Population genetics, Fixation index, Host switching

## Abstract

**Background:**

Specific host-parasite systems often embody a particular co-distribution phenomenon, in which the parasite’s phylogeographic pattern is dependent on its host. In practice, however, both congruent and incongruent phylogeographic patterns between the host and the parasite have been reported. Here, we compared the population genetics of the plateau zokor (*Eospalax baileyi*), a subterranean rodent, and its host-associated flea species, *Neopsylla paranoma*, with an aim to determine whether the two animals share a similar phylogeographic pattern.

**Results:**

We sampled 130 host-parasite pairs from 17 localities in the Qinghai-Tibet Plateau (QTP), China, and sequenced a mitochondrial DNA (mtDNA) segment (~2,500 bp), including the complete COI and COII genes. We also detected 55 zokor and 75 flea haplotypes. AMOVA showed that the percentage of variation among the populations of zokors constituted 97.10%, while the within population variation was only 2.90%; for fleas, the values were 85.68% and 14.32%, respectively. Moreover, the flea *Fst* (fixation index) values were significantly smaller than in zokor. Although the *Fst* values between zokors and fleas were significantly and positively correlated (*N* =105, *R* =0.439, *p* =0.000), only a small amount (*R*^*2*^= 0.19) of the flea *Fst* variations could be explained by the zokor *Fst* variations. The two animals showed very distinct haplotype network structures from each other while co-phylogenetic analyses were unable to reject the hypothesis of an independence of speciation events.

**Conclusions:**

Zokors and fleas have very distinct population genetic patterns from each other, likely due to the influence of other sympatrically-distributed vertebrates on the transmission of fleas.

## Background

Comparative phylogeography aims to compare geographical patterns of evolutionary subdivision across multiple co-distributed species or species complexes, making it a powerful tool for elucidating the demographic and historical nature of intraspecific evolution [1],[2]. Host-parasite systems represent a specific co-distribution phenomenon, in which the parasite’s phylogeographic pattern is typically dependent on their host; comparative investigations of host-parasite systems have thus become one of the most promising directions of comparative phylogeography [3],[4]. Several studies have shown congruent phylogeographic patterns between parasites and their hosts, such as nematodes (*Heligmosomoides polygyrus*) and field mice (*Apodemus sylvaticus*) [5] or mite (*Spinturnix myoti*) and bat (*Myotis punicus*) [6]. In contrast, other studies have shown either incongruent (e.g. lice (*Polyplax arvicanthis*) and rodents (*Rhabdomys* spp.) [7]) or somewhat incongruent (e.g. nematode (*Heligmosomoides* spp.) and mice (*Apodemus* spp.) [8]) phylogeographic patterns between parasites and their hosts. Moreover, many features of the host and its parasite can influence the overall phylogeographic or genealogic congruence, such as the intimacy of the interaction between the two organisms, the scale of vertical transmission of the symbionts through the host generations, and differences in life history traits (population sizes, generation times, and migrating abilities between the hosts and the parasites, etc.) [9]-[12].

Fleas belong to the order Siphonaptera; all species are obligatory blood-feeding parasites of endothermic vertebrates, including mammals and birds [13]. The life cycles of fleas consist of four stages: egg, larva, inactive pupa, and adult. All of these stages are highly dependent on the nests or burrows of their hosts. However, as a typical ectoparasite, all four stages can be viewed as free-living phases. Moreover, adult fleas may also leave their hosts and migrate onto new hosts [13],[14], which potentially negatively influences the congruence of the phylogeographic pattern between fleas and their hosts. Gomez-Diaz *et al.*[15] compared the phylogeography of fleas (*Xenopsylla gratiosa*) and shearwaters (*Calonectris* spp.) and showed that neither genetic distances among host populations nor their spatial distribution explained the patterns of genetic variability observed in the fleas, likely due to a local adaptation to the sympatric host species or to a parasitic exchange during mixing among breeding populations of the hosts. In addition, Jones and Britten [16] compared the genetic structure between prairie dogs (*Cynomys ludovicianus*) and fleas (*Oropsylla hirsuta*); interestingly, the prairie dogs, but not the fleas, showed a significant isolation-by-distance pattern. Moreover, the estimated rates of gene flow among the flea colonies were higher than those among host colonies. Furthermore, these authors argued that other sympatric prairie mammals might be involved in dispersing the fleas, thus resulting in a lack of concordance between the population genetic structures of the host and ectoparasite.

*Neopsylla paranoma* (Ctenophthalmidae) is a Qinghai-Tibet Plateau (QTP) endemic flea [17]. The main host of *N. paranoma* is the plateau zokor (*Eospalax baileyi*), a rodent also endemic to the QTP. During a recent survey (Chen et al., Chinese Journal of Vector Biology and Control, in press), *N. paranoma* comprised 70% of the total fleas collected from trapped zokors. This species is reported as parasitizing sympatric animals (Himalayan marmots (*Marmota himalayana*), hamsters (*Cricetulus longicaudatus*), dipodids (*Allactaga sibirica*), plateau pikas (*Ochotona curzoniae*), and some birds), but comprise a very small proportion (<1%) of the total fleas collected from each of these hosts [18]. It should be mentioned that, as a typical subterranean rodent, plateau zokors spend almost all of their life in underground burrows with rather limited dispersal. Moreover, as a solitary species, zokors of the same gender rarely come into contact with each other; only during the mating season do males and females meet in temporary burrows for a very short time period (several minutes) [19]. Hence, unlike in the shearwaters and prairie dogs mentioned above, zokor-associated fleas have limited opportunities to transfer among zokor populations and between zokors and other sympatric animals. As a result, we hypothesized that the phylogeographic patterns of fleas would largely reflect the phylogeographic history of the zokors. Here, we sampled both zokors and fleas, and using mitochondrial DNA sequences, aimed to establish whether there was a high congruence in the phylogeographic patterns between the two species.

## Results

### General information

**Figure 1:**
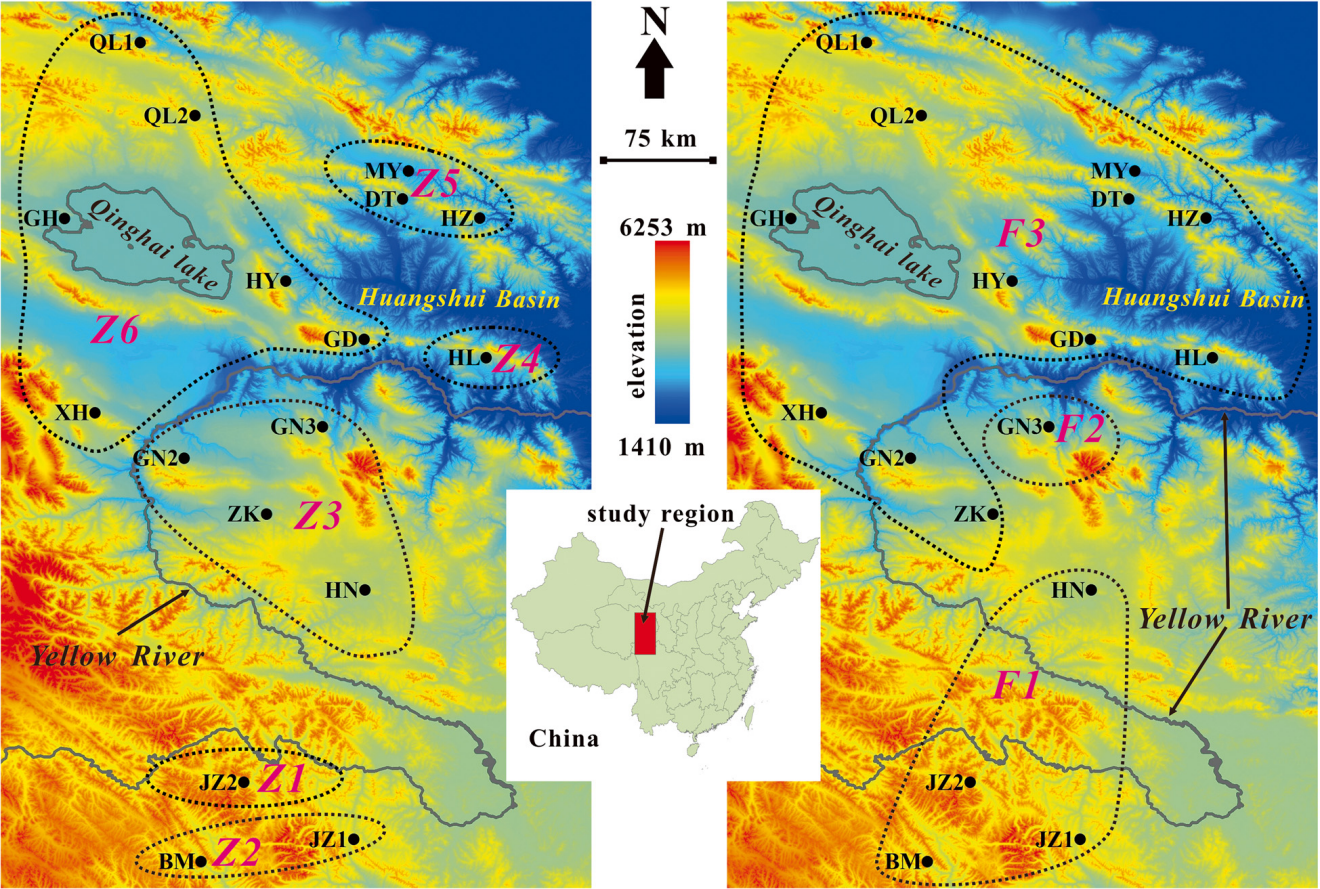
Phylogeographic distributions of phylogenetic clades of zokor (left) and flea (right) populations.

A total of 130 zokor-flea pairs were collected from 17 locations (Table 1, including the GPS coordinates; Figure 1). In 12 locations, 7~12 pairs were collected. In the other 5 locations, only 1~4 pairs were collected because of a low parasitism rate for the fleas. The concatenated mitochondrial DNA (mtDNA) sequences of the zokors were 2,502 bp in length, successively consisting of complete sequences of six genes: tRNA-Cys, tRNA-Tyr, COI (cytochrome c oxidase subunit I), tRNA-Ser, tRNA-Asp, and COII (cytochrome c oxidase subunit II). The main segments were the COI and COII genes, which have 1,545 bp and 684 bp, respectively. The concatenated mtDNA sequences of the fleas were 2,407 bp in length, successively consisting of complete sequences of five genes: tRNA-Cys, tRNA-Tyr, COI, tRNA-Leu, and COII. Moreover, the main segments were also COI and COII genes, which have 1,536 bp and 681 bp, respectively.

**Table 1:** Sampling sites and haplotype distribution of zokors and fleas

**Population**	**County**	**Longitude**	**Latitude**	**Elevation**	**Sample size**	**Haplotype distribution**	
		**/E°**	**/N°**	**/m**		**Zokor**	Flea*
BM	Banma	100.5639	33.12453	3705	7	Zh1~Zh3	Fh1~Fh4
DT	Datong	101.7891	37.15203	2988	12	Zh4~Zh11	Fh5~Fh13
GD	Guide	101.5539	36.29937	3119	1	Zh12	Fh14
GH	Gonghe	99.73498	37.03382	3209	10	Zh13, Zh14	Fh15~Fh22
GN2	Guinan	100.4618	35.57738	3306	10	Zh15~Zh20	Fh23~Fh26
GN3	Guinan	101.3012	35.76745	3302	4	Zh21	Fh27~Fh29
HL	Hualong	102.2971	36.18848	3185	10	Zh22~Zh26	Fh30~Fh37
HN	Henan	101.5598	34.77488	3552	2	Zh27, Zh28	Fh38
HY	Huangyuan	101.0782	36.65460	3043	10	Zh29~Zh35	Fh39~Fh48
HZ	Huzhu	102.2560	37.03535	2857	10	Zh36~Zh38	Fh49~Fh55
JZ1	Jiuzhi	101.4916	33.25988	3741	8	Zh39	Fh56~Fh58
JZ2	Jiuzhi	100.8221	33.60497	3847	4	Zh40, Zh41	Fh59~Fh61
MY	Menyuan	101.8255	37.32348	2715	8	Zh42, Zh43	Fh62
QL1	Qilian	100.1932	38.10408	3213	10	Zh44~Zh46	Fh63~Fh66
QL2	Qilian	100.5255	37.65958	3566	12	Zh47~Zh52	Fh67~F69, Fh64
XH	Xinghai	99.9187	35.85298	3566	11	Zh53, Zh54	Fh70~Fh74
ZK	Zeku	100.9617	35.23765	3428	1	Zh55	Fh75

*Fh25 was shared by GN2, GN3, and XH; Fh64 was shared by QL1 and QL2.

From the 130 zokor sequences, a total of 287 variable sites and 55 haplotypes (Zh1~Zh55) were detected. No haplotype was shared by two or more populations. For the 130 flea sequences, a total of 157 variable sites and 75 haplotypes (Fh1~Fh75) were detected. Of these, there were two haplotypes shared by two (Fh64, shared by QL1 and QL2) and three (Fh25, shared by GN2, GN3, and XH) populations, respectively. The distributions of the haplotypes are listed in Table 1.

### Population structure

AMOVA (Analyses of Molecular Variance, based on all 17 locations) showed that, for zokors, the percentage of variation between populations constituted 97.10%, while the within population variation constituted only 2.90%. For fleas, the percentage of variation among populations and within populations constituted 85.68% and 14.32%, respectively. All these AMOVA tests were highly significant (*p* = 0.000).

The *Fst* (fixation index) values and geographic distances among the 15 zokor/flea populations are listed in Table 2. A Mantel test showed that the correlation of (*R*^*2*^) the zokor *Fst* with the geographic distance was 15.84% (*P* = 0.000), while the flea *Fst* values were also significantly correlated with geographic distance (*p* = 0.000). However, the *R*^*2*^ value was 46.20%, much higher than that of the zokors. A Wilcoxon Signed Ranks Test revealed that the flea *Fst* values were significantly smaller than zokor *Fst* (*N* =105, *Z* = −7.712, *p* =0.000). In addition, a Spearman correlation analysis showed that the *Fst* values of zokors and fleas were also significantly and positively correlated (*N* =105, *R* =0.439, *p* =0.000).

**Table 2:** **Geographic distance (upper-right triangular) and genetic distance (*Fst*) (lower-left triangular; zokor, before the comma; flea, after the comma) among 15 populations (for the other 2 which had only one sample each were not considered)**

	**BM**	**DT**	**GH**	**GN2**	**GN3**	**HL**	**HN**	**HY**	**HZ**	**JZ1**	**JZ2**	**MY**	**QL1**	**QL2**	**XH**
BM	—	460.59	440.12	272.21	300.90	375.26	204.91	394.41	460.57	87.80	58.45	479.83	553.40	503.09	308.34
DT	0.98,0.94	—	183.07	211.48	159.78	116.23	264.66	84.05	43.50	432.79	403.28	19.31	176.10	125.26	221.02
GH	0.99,0.96	0.98,0.63	—	174.25	198.68	247.67	299.89	126.95	224.30	448.18	392.96	188.39	125.45	98.61	132.02
GN2	0.98,0.98	0.97,0.71	0.98,0.40	—	78.86	179.09	133.90	131.77	228.37	273.95	221.26	229.11	281.36	231.09	57.87
GN3	0.99,0.77	0.98,0.78	1.00,0.77	0.39,0.80	—	101.28	112.63	100.47	164.77	278.77	243.89	178.98	277.43	221.11	125.29
HL	0.97,0.97	0.96,0.60	0.96,0.68	0.97,0.81	0.97,0.79	—	170.60	120.98	94.11	333.31	316.82	132.88	283.10	227.13	217.61
HN	0.97,0.90	0.97,0.93	0.99,0.95	0.43,0.99	0.66,0.53	0.96,0.96	—	213.10	258.68	168.21	146.52	283.90	389.20	333.33	191.24
HY	0.96,0.92	0.94,0.47	0.62,0.12	0.96,0.26	0.95,0.72	0.91,0.51	0.94,0.90	—	113.27	378.55	339.14	99.70	178.92	121.85	136.98
HZ	0.99,0.95	0.63,0.15	0.99,0.65	0.98,0.75	0.99,0.77	0.97,0.64	0.98,0.94	0.95,0.48	—	424.80	402.39	49.87	217.46	168.28	247.24
JZ1	0.84,0.67	0.98,0.95	1.00,0.97	0.98,0.99	1.00,0.79	0.98,0.97	0.99,0.96	0.97,0.93	0.99,0.96	—	73.09	452.00	550.14	495.96	321.81
JZ2	0.99,0.42	0.98,0.94	1.00,0.95	0.98,0.99	1.00,0.68	0.98,0.96	0.98,0.92	0.96,0.91	0.99,0.95	1.00,0.62	—	422.55	502.34	450.62	262.70
MY	0.98,0.99	0.59,0.53	0.99,0.83	0.98,0.96	0.99,0.80	0.96,0.86	0.98,1.00	0.94,0.65	0.84,0.62	0.99,1.00	0.99,0.99	—	167.99	120.87	236.08
QL1	0.98,0.98	0.97,0.47	0.86,0.80	0.98,0.90	0.98,0.82	0.95,0.80	0.97,0.98	0.46,0.63	0.98,0.53	0.99,0.98	0.99,0.98	0.97,0.69	—	57.34	250.94
QL2	0.98,0.93	0.96,0.28	0.76,0.49	0.97,0.59	0.98,0.76	0.94,0.54	0.97,0.91	0.25,0.33	0.97,0.29	0.98,0.94	0.98,0.92	0.97,0.37	0.61,0.19	—	207.62
XH	0.99,0.97	0.98,0.70	0.96,0.41	0.99,0.14	1.00,0.79	0.96,0.77	0.99,0.97	0.70,0.28	0.99,0.73	1.00,0.98	1.00,0.97	0.99,0.91	0.89,0.87	0.84,0.58	—

### Network and co-phylogenetic results

**Figure 2:**
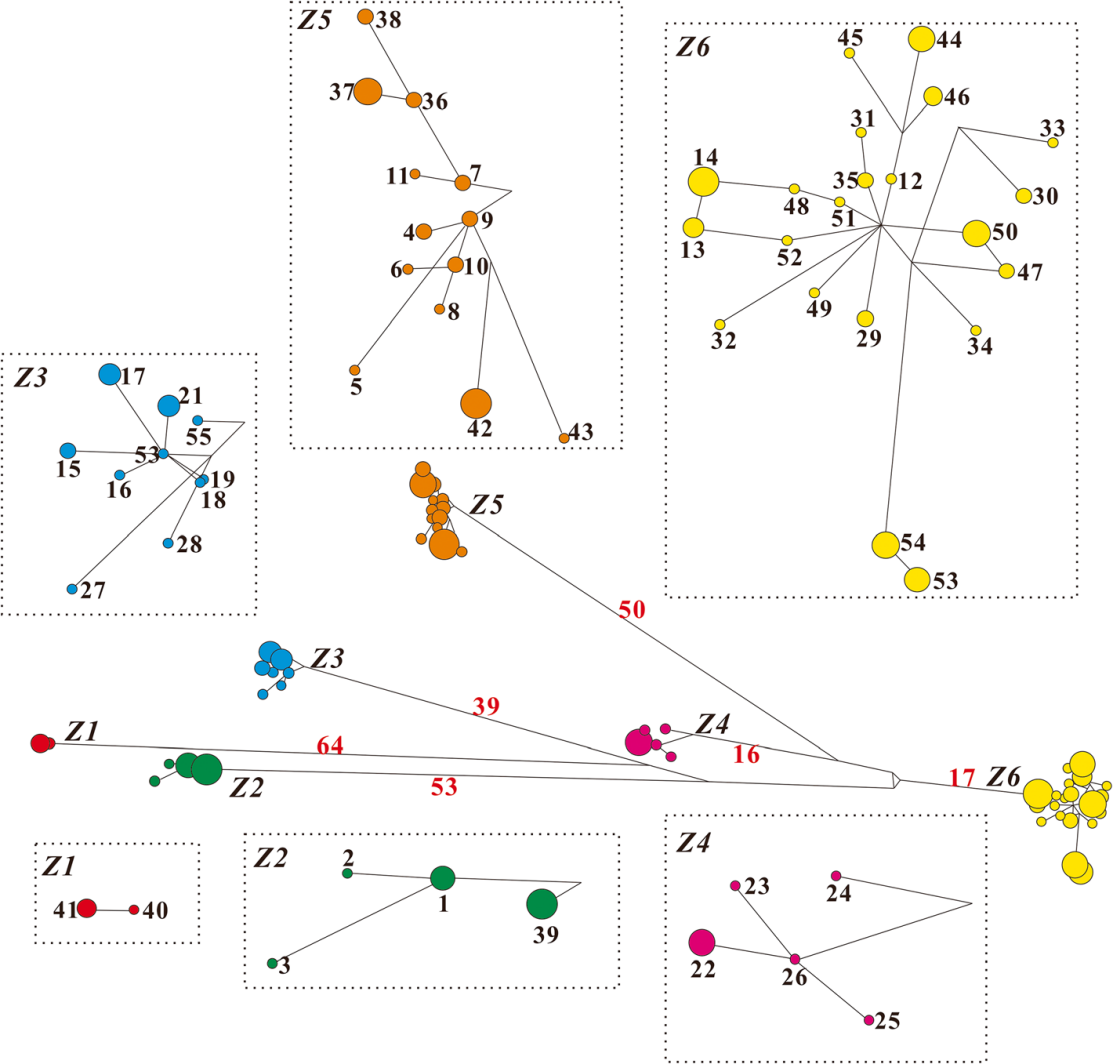
**Network structures of zokor haplotypes.** Z1~Z6 show the network clades, also see Figure 1 for details. The red numbers show the lengths (number of substitutions) of the main branches.

**Figure 3:**
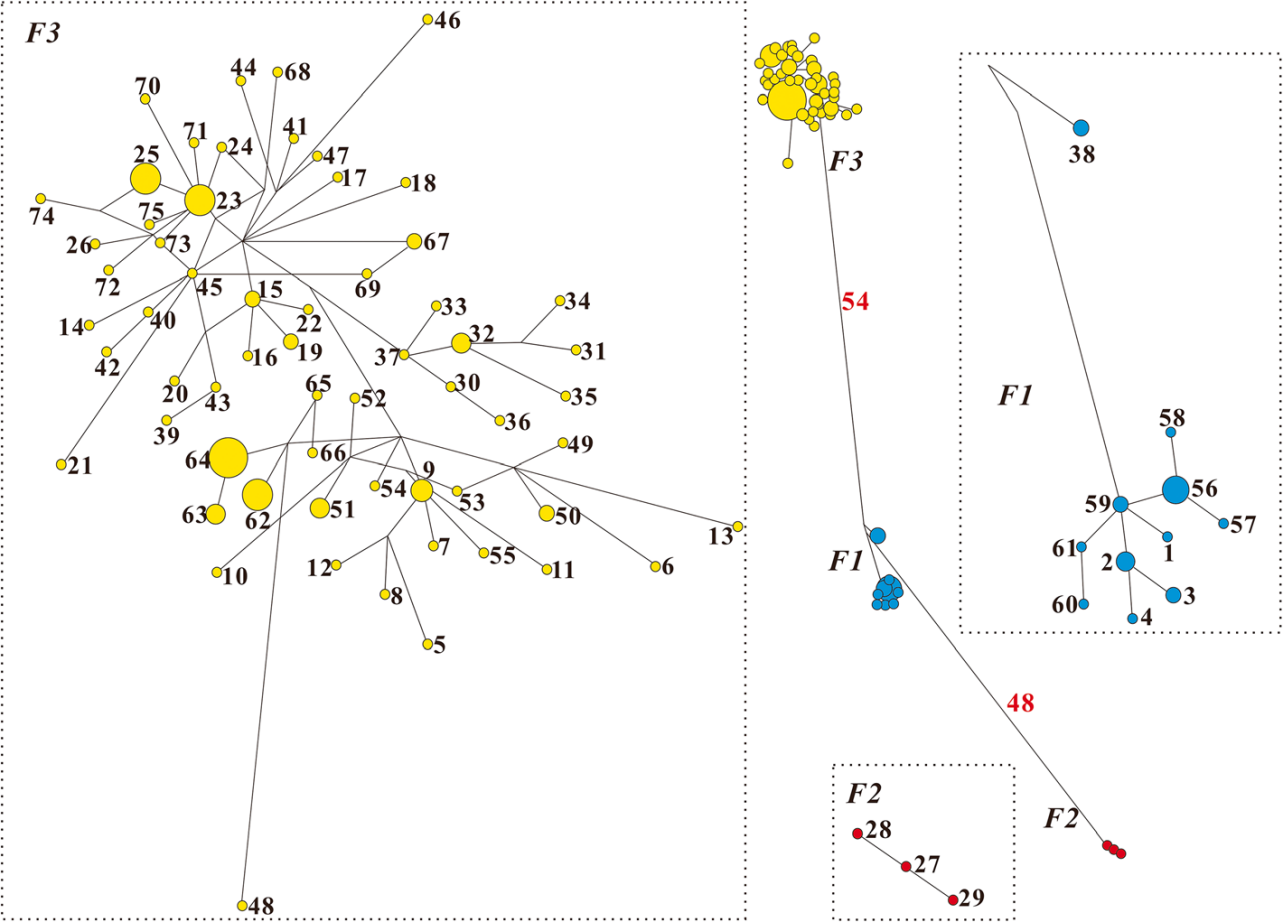
**Network structures of flea haplotypes.** F1~F3 show the network clades, also see Figure 1 for details. The red numbers show the lengths (number of substitutions) of the main branches.

Both zokors and fleas had very distinct network features. The 55 zokor haplotypes formed into six distinct clades (Z1~Z6, Figure 2), corresponding to six geographic regions (Figure 1). However, the 75 flea haplotypes formed only three clades: F1 included four populations while F2 included only one population and F3 included the other 12 populations related with the majority of the haplotypes (61 of 75) (Figures 1 and 3).

Co-phylogenetic analyses, based on either the 17 populations (ParaFitGlobal = 0.0156, *p* = 0.286) or the 15 populations (ParaFitGlobal = 0.0107, *p* = 0.284), were unable to reject the hypothesis of independence of speciation events. Based on ParaFit1 values, only one (JZ2) of 17 or 15 host-parasite links was significant (*p* <0.05).

## Discussion

### Incongruent genetic structure

Our results showed that the zokors and fleas had very distinct population genetic structures from each other. First, the AMOVA results revealed that the variation between the flea populations (85.68%) was considerably lower than that among zokor populations (97.10%). Second, flea *Fst* values were significantly smaller compared to the zokor *Fst* (*p* =0.000). Third, each zokor haplotype was uniquely distributed within a single population; for fleas, however, two haplotypes were shared between two or more populations. Fourth, the co-phylogenetic analyses were unable to reject the hypothesis of independence of speciation events between the two animals.

The results of the network analyses also showed a distinct geographic haplotype distribution pattern between the two species. The JZ2 population, which was an independent clade in zokors, showed a closer relation with its neighboring populations, JZ1 and BM. Also, the 12 populations (DT, GD, GH, HL, GN2, HY, HZ, MY, QL1, QL2, XH, and ZK), which were grouped into four separate clades in zokors, were grouped into a single clade in fleas (Table 1 and Figure 1). These results indicate that fleas have a lower level of genetic differentiation than zokors.

### Effects of geographic barriers

The QTP is the largest and highest plateau on Earth. The QTP began a severe uplift during the Pliocene, and the uplift continued until the Quaternary glaciations. As a result, the plateau began to topographically diversify due to the intricate and continual development of new mountains and watercourses [20], which likely became geographical barriers that helped create various genetic structures for endemic animals [21]. It should be mentioned that with different dispersion abilities, each species of animal living on the QTP tends to have very distinct genetic structures. For example, there is a reported absence in distinct genetic structures among populations of a snow finch (*Pyrgilauda ruficollis*), which is a strong flier and tend to move seasonally. In contrast, a high level of genetic structure was reported for the ground tit (*Pseudopodoces humilis*; a resident bird species) and a lizard (*Phrynocephalus vlangalii*), because the two species tend not to disperse (reviewed in [22]).

Like other subterranean rodents (such as the tiny tuco-tuco (*Ctenomys minutus*) [23]), geographic barriers seem to have played an important role in shaping the phylogeographic structure of zokors: the Yellow River separated Z1 and Z2 from Z3~Z6; the extremely high altitude at and around the JZ2 group isolated the haplotypes in this region into a basal clade Z1, whereas the Huangshui Basin separated Z4, Z5, and Z6 from each other. However, it is noteworthy that distance, rather than physical barriers, seems to have played an important role in shaping the geographic patterns of fleas: the *R*^*2*^ of the IBD effects in fleas (46.20%) was much higher than that of the zokors (15.84%). The most evident example of the different effects of geographic barriers on population genetics occurred between the XH and GN2 groups. The geographic distance between the two populations was only 57.87 km, but the *Fst* between them was 0.99; as such, they were grouped into two different clades Z3 and Z6. For fleas however, the *Fst* between the two populations was only 0.14, and subsequently, they were grouped into a monophyletic clade F3. Taken together, these results suggest that the QTP-associated geographic barriers had a strong isolation effect in the genetic variation of zokors; in contrast, this effect was much weaker on fleas.

### The third-part flea hosts?

As mentioned above, the differences in life history traits, such as population sizes, generation times, and migrating abilities, between the hosts and the parasites could directly or indirectly influence the interactions between the two organisms [10]-[12]. Although little is known about the ecology of the flea (*N. paranoma*), much more is known about zokors. Zokors spend most of their lives underground and have a very limited migration ability (only about 66.9 meters per year) [24]. As such, genetic interactions between the populations resulting from zokor migrations would also be very limited. In the current paper, we found that no haplotype was shared by different populations of zokors, further supporting this view. Moreover, zokors live a solitary life except during the mating season [19]. As a result, the migration of fleas via zokor contact should also be very limited. In other words, even if the fleas in our study have very distinct life history traits compared to the zokors that they live on, the phylogeographic features of fleas should still largely depend on the zokors, provided no other vertebrates were involved in this host-parasite system.

Unexpectedly, however, we found that the fleas had lower inter-population genetic variation levels than zokors, suggesting that some other host animals have also mediated their migration and subsequently had a significant influence on their phylogeographic patterns. The degree of association between fleas and hosts varies among different host/flea species [25]. Zhang [18] reported that Himalayan marmots, hamsters, dipodids, plateau pikas, and some birds, all of which are good migrators, could also be infected by *N. paranoma.* It is puzzling, however, how zokors might exchange their parasites with above-ground animals. Pikas are the most closely related sympatric animals to plateau zokors. In many cases, pikas occupy the burrows of zokors [26], which might provide opportunities of flea exchange between the two species. Moreover, as a key species in the QTP [27], plateau pikas also share habitats with most of the other species in the QTP, as well. We hypothezed that the plateau pikas first serve as a mediator of fleas between zokors and above-ground animals (including birds), and then these above-ground animals help to disperse the fleas among different regions. It should be mentioned that there is a haplotype (Fh25) that is shared by GN2, GN3, and XH, which were separated by the Yellow River. Since a large majority of mammalian animals seem unable to migrate over the Yellow River, we suggest that birds might play a more important role for cross-river flea transmission.

## Conclusions

Despite the unique subterranean lifestyle of the zokor, the population genetic patterns of the fleas were distinct from their hosts. Moreover, fleas had a lower level of genetic variation between populations compared to zokors, suggesting other sympatrically distributed vertebrates serve as a mediator for flea transmission.

## Methods

### Sampling

During April and May, 2012, individual zokors were sampled using ground arrows [28] from the east of Qinghai Province, China. The geographical information at each sampling site was recorded using an Etrex GPS unit (Garmin, Taiwan). Each of the trapped zokors was immediately put into a plastic bag filled with diethyl ether for ten minutes. The dead fleas were collected and stored in 75% alcohol. Muscle samples from each zokor were collected and fixed in 95% alcohol. The taxonomy of the fleas was identified under light microscopy. All animal work in this study was conducted with ethical approval from the Ethics Committee, Northwest Institute of Plateau Biology, Chinese Academy of Sciences. No specific permissions were required for the locations/activities in this study. The field studies did not involve endangered or protected species.

### DNA extracting, amplifying and sequencing

Total DNA of each zokor sample was extracted using standard methods for animal tissue [29]. The sequences of the three mitochondrial DNA (mtDNA) segments were amplified by polymerase chain reaction (PCR) using three pairs of primers (Table 3) designed with reference to the plateau zokor mitochondrial genome (accession No. JN540033.1). Total DNA from each flea was extracted using a spin column kit (DNeasy tissue kit; Qiagen, Germany), according to the manufacturer’s protocol. The sequences of three target mtDNA segments were amplified using three primer pairs (Table 3). Two were designed according to the mitochondrial genome of a mecopteran species (*Boreus elegans*, NC_015119.1), which was viewed as the closest sister group of the fleas [30], and the other one is according to Whiting [30].

**Table 3:** Primers used for amplifying and sequencing mitochondrial segments of zokors and fleas

**Primer pairs**	**Forward (5′→3′)**	**Reverse (5′→3′)**
zokor1-F/R	CCTCAACAAACAACATAA	TGAAAAGGAAAATAAAAC
zokor2-F/R	TTATTTCCCATATCGTTAC	GTTCTCCTGGTTTTAGTTC
zokor3-F/R	CAACAAACCTGGAATGAC	GAGAAAGAGGCGAATAAA
flea1-F/R	ACGCCCTTTCATTTTTGA	AAGTTTACCTGATTCTTGAG
flea2-F/R	TGGTCACCCAGAAGTA	AGAAGGAAGGGCAAT
COII-F-leu/R-lys*	TCTAATATGGCAGATTAGTGC	GAGACCAGTACTTGCTTTCAGTCATC

*According to Whiting (2002) [30].

PCR amplifications were performed in total reaction volumes of 50 μL, containing 10mM Tris–HCl (pH 8.3), 1.5 mM MgCl_2_, 50 mM KCl, 100 μM of each dNTP, 0.25 μMol each primer (synthesized by Sangon Ltd., China), 0.5 μl of template DNA, and 2.5U Taq DNA polymerase (Takara). The reaction mixtures were denatured at 95°C for 5min and subjected to 35 cycles of 45 s at 95°C, 1 min at 50~55°C (depending on the primer pair), 1.5 min at 72°C, and a final extension step of 7 min at 72°C. PCR products were purified using a CASpure PCR Purification Kit, following the manufacturer’s recommended protocol (Casarray, Shanghai, China). Purified DNA products were sequenced in both directions with the PCR primers on an ABI 373 automated sequencer. Both strands of each segment were sequenced using forward and reverse primers. Sequences were recorded in both strands with an overlap of at least 30%. The sequences of each segment were checked by eye and aligned using CLUSTAL W [31] and refined manually. Finally, the three segments of each species were combined for further analyses.

### Genetic variation calculation

Basic polymorphism data (variable sites, haplotype distribution, etc.) were determined using DnaSP (version 5) [32]. The Arlequin (version 3.11) [33] software was used to calculate AMOVA (Analyses of Molecular Variance) and *Fst* values among populations. We then used ArcGIS software (version 9.3) to calculate the geographic distances among the sampling locations, and used Arlequin to carry out Mantel test [34] to examine isolation-by-distance effects of the two species (1000 permutations were used). We used a non-parametric 2 Related Samples Test (Wilcoxon Signed Ranks Test) in SPSS software (version 20.0) to compare the zokor/flea *Fst* values. We also used a Spearman correlation in SPSS to test the relationships between *Fst* values of zokors and fleas. It should be mentioned that, in two locations (GD and ZK) there was only one zokor-flea pair each, which could induce unrealistic *Fst* estimates such as negative values. Hence, for the analyses relating *Fst* values, these locations were excluded.

### Network and co-phylogenetic analysis

Roehl data files were generated with DnaSP. The haplotype networks of zokors and fleas were constructed with the program NETWORK (version 4.6.1.2). The Median Joining method [35] was used to calculate network structure. Then the drawn networks were saved as bitmap and were introduced into CorelDraw (version X6) to prepare the final feature figures.

The optimized versions of Pierre Legendre’s Parafit (i.e. AxParafit) [36] was used to test the co-phylogenetic congruence between zokors and fleas. First, the between group mean distance (Kimura 2-parameter model) matrices based on the 17 populations and 15 populations (GD and ZK excluded) were calculated with MEGA (version 5.2) [37]. The global-fit congruence and individual host-parasite interaction analysis were tested with 100,000 permutations using zokor distance matrix, flea distance matrix, and the zokor-flea association file. Each individual zokor-flea interaction is determined to be significant if either its ParaFit 1 or Parafit 2 p-value <0.05.

### Availability of data

The data supporting the results of this article are available in the Dryad repository (http://dx.doi.org/10.5061/dryad.d0v11) [38]. The haplotypes are also available in GenBank (accession numbers: Zh1~Zh55, KJ470899~KJ470953; Fh1~Fh75, KJ470954~KJ471028).

## Abbreviations

QTP: The Qinghai-Tibet Plateau

mtDNA: Mitochondrial DNA

COI: Cytochrome c oxidase subunit I

COII: Cytochrome c oxidase subunit II

AMOVA: Analyses of molecular variance

PCR: Polymerase chain reaction

cytb: Cytochrome b

*Fst*: (Fixation index)

## Competing interests

The authors declare that they have no competing interests.

## Authors’ contributions

GL, JS, and TZ conceived the study. FZ carried out the molecular genetic work. GL and JS performed data analysis. HC and XD coordinated sampling of material. GL and JS interpreted the biological context of results. All authors wrote, read and approved the final manuscript.
